# Social prescription for isolated parenting in Japan: Socioeconomic characteristics of mothers with weak social connectivity in their community

**DOI:** 10.1111/hsc.13610

**Published:** 2021-10-17

**Authors:** Hikaru Honda, Toshiko Kita

**Affiliations:** ^1^ School of Nursing Sapporo City University Sapporo Japan

**Keywords:** child maltreatment, Japan, motherhood, mothers, neighbourhood interaction, social connectivity, social isolation

## Abstract

The social connection of mothers is important for the sound development of children and the prevention of child maltreatment. Understanding the attributes of mothers at risk of isolation enables community workers to support vulnerable mothers. This cross‐sectional study aimed to identify the socioeconomic predictors of isolation risk for mothers and was conducted in Japan between December 2018 and February 2019. The self‐administered questionnaire included the Social Connectivity of Mother Scale along with maternal age, marital status, employment status, education, number of children, years of child‐rearing experience, whether childcare or kindergarten was used, family structure, years of residence, housing type, family finances, and level of neighbourhood interactions. There were 510 valid responses (51.6%). In the multiple regression analysis, five socioeconomic characteristics were associated with mothers’ low social connectivity: the standardised coefficient of the maternal age of 20–24 was −0.12 (*p* = 0.004), lack of childcare or kindergarten usage, −0.09 (*p* = 0.032), and poor family finances, −0.09 (*p* = 0.031); mothers’ perception of neighbourhood interactions was found to be poor at −0.29 (*p* < 0.001). The model did not take into account the effects of family finances, and the scores were low when the highest level of education of the mother was junior high or high school. Mothers’ perception of neighbourhood interactions was a significant predictor of isolation risk, along with maternal age, education level, and financial comfort. Our findings give policymakers, community workers, and community leaders an insight into the importance of cultivating interactions among neighbourhood communities.

**What is known about this topic?**

- Isolated parenting has become a serious problem for Japanese mothers.
- Preventing mothers from feeling isolated protects their mental health and ensures the healthy development of children.
- The socioeconomic characteristics that affect mothers' weak social connections are not yet clear.

**What this paper adds?**

- When mothers are not aware of neighbourhood connections and interactions, and of helpful key community persons, support for raising awareness of their value is useful.
- Supporting younger mothers, particularly those aged 20–24, is especially important as they are less able to connect with people.
- Policies focused on reducing economic inequality are necessary, because lower economic resources are associated with fewer social connections.

## 1 INTRODUCTION

Social isolation is currently a global concern, with research in the field of public health being conducted in many countries (Leigh‐Hunt et al., 2017). Isolation has been reported to exacerbate not only depression (Quach & Burr, 2020) and cardiovascular diseases (Leigh‐Hunt et al., 2017; Valtorta et al., 2016) but also frailty in the elderly (Gale et al., 2018). Matthews, Nieh, et al. (2016) found a change in the functional role of the dopamine neurons, which may mediate social reward when individuals experience social isolation. Cacioppo et al. (2015) report that chronic social isolation increases the activation of the hypothalamic pituitary adrenocortical axis; these neurobiological mechanisms may contribute to one's health conditions. Matthews, Danese et al. (2016) revealed the negative impact of social isolation on the mental health of young people. Nowadays, social isolation is a common problem among all generations.

According to Carlos et al. (2019), with respect to child‐rearing, the parents of abused children have homogeneous social connections that lack diversity and density, suggesting the importance of parents having rich social connections. Social support gained through parents’ social connections is also important from the perspective of its contribution to child protection and the prevention of child maltreatment (Thompson, 2015). McDonald et al. (2018) found that a nurturing environment, developed through maternal social connections, affects the positive development of children in the future. Therefore, mothers’ social connections are important for the sound development of children as well as the prevention of child maltreatment.

However, in the current social environment surrounding child‐rearing in Japan, it may not be easy for mothers to become socially ‘connected.’ First, from the perspective of population structure, the birth rate continues to decline (Japan Cabinet Office, 2019a), and there are fewer opportunities for parents to meet peers in the neighbourhood. In addition, with the increasing prevalence of nuclear families, many people live far away from grandparents, and support from relatives is not expected as much as it once was (Kudo, 2013). Furthermore, in multi‐dwelling units, improved structural aspects and security measures have led to decreased casual conversations with neighbours (Tanaka et al., 2011). Work‐wise, long hours and non‐regular employment are becoming a problem, widening the economic gaps within the child‐rearing generation (Japan Ministry of Health, Labour, & Welfare, 2017). The diversification of lifestyle and individual values (Japan Ministry of Health, Labour, & Welfare, 2018) and the spread of individualism make it all the more difficult to interact with people (Ogihara & Uchida, 2014). Therefore, modern mothers have to be even more proactively involved and ingenious to gain ‘connections.’

Additionally, Japanese child‐rearing practices are influenced by parents’ cultural background. Many mothers are forced to resign from their jobs or take lengthy parental leaves to undertake their child‐rearing responsibilities almost single‐handedly, resulting in social isolation (Akiba et al., 2013). Sometimes mothers are exposed to evaluations of their role as parents by others (Hososaka & Kayashima, 2017). They are at high risk of being psychologically isolated, thinking that they have to discipline their child by themselves.

Cases supported by public health nurses often involve children with developmental or health issues or instances where parents have mental or physical vulnerabilities (Yoshioka‐Maeda & Kuroda, 2017). Mothers in such situations are known to be more isolated (Currie & Szabo, 2020). Understanding the attributes of mothers at risk of isolation enables public health nurses to build support for vulnerable mothers from a wellness perspective. Cotterell et al. (2018) studied the relationship between isolation and socioeconomic factors, but the report limits its focus to older adults. Additionally, Phuphaibul et al. (2014) reported on the relationship between child development and parental socioeconomic factors. However, prior studies reporting the impact of socioeconomic characteristics on maternal social connections are not apparent.

In light of these considerations, the purpose of this study was to identify the characteristics of mothers with low capability for connection within the community. The clinical significance of this research is that it seeks to not only investigate factors related to social isolation within the demographic of vulnerable mothers but also study it as a social issue from the perspective of community development. By understanding the underlying processes and factors, it might be possible to provide an insight to policy makers and community leaders, so that they can develop measures that promote interactions among neighbourhood communities.

## 2 METHODS

### 2.1 Research participants

Questionnaires were sent to the target audience of 1,000 mothers of infants. However, 12 questionnaires were not delivered because of duplicate or misaddressed participants. To calculate the sample size, G*power, provided by Heinrich Heine University Düsseldorf, was used. The calculation method assumed linear multiple regression, calculated with an effect size 0.02, α error 0.05, and power of test 0.8. As a result, the required sample size was 647. Since the response rate of the survey of mothers in Japan is reported to be 68% (Yamaoka et al., 2016), the ideal research participant number was set for 1,000 mothers. The survey was conducted in the Japanese city of ‘*A*’. The annual number of births in City *A* was 12,561 (as of April 2020).

The reason for studying mothers of infants is that public health nurses’ support within the Japanese maternal healthcare system is primarily provided during infancy.

### 2.2 Data collection methods

Participants were recruited using a stratified randomisation method on the Basic Resident Registration system managed by City *A*. Five wards were extracted using a random number table of City *A*'s 10 wards. Districts within each ward were randomly selected. For this study, 200 participants were recruited from each ward.

The survey was conducted via an anonymous self‐report questionnaire method using return‐post. The study period was from December 2018 to February 2019.

### 2.3 Questionnaires

#### 2.3.1 Socioeconomic characteristics of mothers

Details were sought on the following individual attributes of socioeconomic characteristics affecting mothers: maternal age, marital status, employment status, highest level of education, number of children, years of child‐rearing experience (age of the oldest child), whether or not childcare or kindergarten was used, family structure, years of residence, type of housing (multi‐dwelling unit or single‐detached dwelling), family finances, and mothers’ perception of interactions within the neighbourhood.

The mothers’ subjective impressions of family finances (The Japan Institute for Labour Policy & Training, 2015) and perception of interactions in the neighbourhood (Japan Cabinet Office, 2007) were investigated using the following items: ‘Financially, my family is… (comfortable, somewhat comfortable, not very comfortable, not comfortable)’, and ‘In the community I live in, the level of neighbourly interaction with other child‐rearing parents is… (apparent, somewhat apparent, not very apparent, not apparent)’.

#### 2.3.2 Social connectivity of mothers with people in the community scale

When children are born, mothers develop new relationships in the region through their role, in addition to their existing friendships. Unlike the fixed relationships of schools and corporate organisations, mothers meeting up with others is largely reliant on their freedom and discretion. Therefore, they need high levels of proactivity to make community connections.

Prior studies featured scales for measuring common interpersonal skills but not mothers’ abilities, reflecting the typical characteristics of the child‐rearing culture in Japan. Thus, in earlier research, the authors developed a scale to measure mothers’ ability to connect with the people in the community through child‐rearing, called Social Connectivity of Mother Scale (Honda et al., 2020). However, this scale does not measure the structure of the connection as a social network. Holt‐Lunstad (2018) explains that social connection comprises elements such as a sense of connection to others that is based on positive and negative qualities. Similarly, this scale measures not only the skill to develop favourable connections with others but also the ability to convert the connections that the mother already has as a form of social support.

The ability to connect with people in the community was assessed using four subscales (Table 1). The scale consists of 17 items, each rated on a four‐point Likert scale ranging from *Strongly Agree* (3) to *Disagree* (0). The reliability coefficient of the scale in this research was Cronbach's α = 0.84. The scales indicated sufficient criterion‐related validity not only against the presence of mothers’ social isolation but also against the WHO‐5 Well‐being Index (Awata et al., 2007) that measures the mental health of mothers, and the UCLA Loneliness Scale Version 3 (Russell, 1996). Thus, the reliability and validity of the scale have been verified (Honda et al., 2020). In this study sample, the Confirmatory Factor Analysis results indicators for the degree of conformance of the model were 0.922, 0.895, 0.910, and 0.065 for GFI, AGFI, CFI and RMSEA, respectively.

**TABLE 1:** Social connectivity of mother with people in the community scale (*N* = 510)

Items		Mean ± *SD*
Total scores of the whole scale (α = 0.84)		31.5 ± 6.63
Factor 1: Confidence in interacting with people (α = 0.81)		7.8 ± 2.88
1	I can create good relationships with almost anyone	1.7 ± 0.76
2	Usually I can find a like‐minded person, no matter the situation	1.6 ± 0.76
3	Usually I am the one who initiates a conversation with someone else	1.4 ± 0.84
4	Even if I meet someone new, I am able to enjoy the light‐hearted relationship of that situation	2.0 ± 0.61
5	With like‐minded people, it is usually me who suggests we exchange contact details	1.1 ± 0.82
Factor 2: Positive feelings toward social connectivity (α = 0.63)		9.6 ± 2.32
6	The people in my neighbourhood care about me and my children	1.5 ± 0.87
7	I think that the area where we live is a safe environment to raise children	1.9 ± 0.66
8	Staff members of public organizations^a^ care about me and my children	1.9 ± 0.78
9	I am happy if someone in the neighbourhood greets me	2.3 ± 0.65
10	When I talk with other mothers, I feel relieved that my parenting ‘is actually fine’	1.9 ± 0.69
Factor 3: Interest in interacting with people (α = 0.72)		7.3 ± 2.26
11	Through raising children, I feel that I want to contribute something to the community	1.7 ± 0.77
12	I would like to interact more with people in the community through my children	1.6 ± 0.75
13	I want to participate in child‐related community events as much as possible	2.0 ± 0.74
14	I am interested in government measures relating to parenting	2.0 ± 0.79
Factor 4: Kindness toward other parents & children (α = 0.77)		6.9 ± 1.58
15	I would like to be kind to other people in my neighbourhood who are raising children	2.4 ± 0.59
16	I would like to treat other children kindly, just as I treat my own	2.4 ± 0.60
17	I would like to teach other children ‘the importance of following the rules,’ just as I would with my own	2.1 ± 0.72

The range of total scores: Minimum 12.0/Maximum 51.0. 95% CI, mean of total scores: 30.9–32.1.

^a^ Public organisations mean health centres, child‐rearing support centres, daycare/nursery, etc.

### 2.4 Analysis method

The normality of scores on mothers’ social connectivity with people on the community scale was confirmed using the Shapiro–Wilk test. For ease of interpretation, scores regarding family finances and neighbourhood interaction were reorganised and analysed as two groups, ‘high’ and ‘low’. A *t*‐test, one‐way ANOVA, and multiple comparison procedures were used to test for differences in the socioeconomic characteristics of the mothers.

Following this process, first, stepwise multiple regression analysis was conducted, with scale scores as the dependent variable, to explore the factors behind mothers’ weak ability to connect with people in the community. As a second step, to verify the interaction, multiple regression analysis by forced entry was performed. The respective model was presented after confirming that multicollinearity did not affect the analysis. IBM SPSS Statistics version 25 was used in the above analysis, with a significance level of 5%.

### 2.5 Ethical considerations

The questionnaires contained a written explanation that the responses would be anonymous and voluntary, and nonparticipation would not incur any adverse effects. Furthermore, using the Basic Resident Register to extract participants was carried out per the formal procedures set forth in the ordinances of City *A*. This research study was conducted with the approval of the Ethics Committee of the Sapporo City University (No. 1812–1) to which the authors are affiliated.

## 3 RESULTS

Responses were received from 525 (53.1%) respondents out of the 988 distributed questionnaires. The 510 responses that had no missing values regarding socioeconomic characteristics (51.6% effective response rate) were analysed. There was no significant difference (*p* = 0.497) in scale scores between the groups of effective responses and non‐effective responses.

### 3.1 Summary of the individual attributes of mothers

Table 2 summarises the individual attributes of the mothers. Mothers aged 30–34 were the most common age group, with 190 people (37.3%). Those under 20 were not included. Regarding employment status, homemakers (unemployed) were the largest group, at 235 (46.1%), followed by 196 (38.4%) working mothers. Most mothers (455; 89.2%) had more than 1 year of child‐rearing experience (the oldest child's age). There were 479 (93.9%) nuclear family households consisting of only parents and children. A total of 297 mothers (58.2%) lived in multi‐dwelling units, 290 (56.9%) were from financially comfortable families, and 315 (61.8%) indicated that neighbourhood interactions were prevalent.

### 3.2 Relationships between mothers’ social connectivity and socioeconomic characteristics

Table 2 shows the relationships between mothers’ social connectivity with people in the community and socioeconomic characteristics. Mothers aged 20–24 had the lowest mean scale scores on social connectivity (*M* = 24.1 ± 5.9), and ANOVA results indicated *p* = 0.005. Results from a multiple comparison procedure between the group aged 20–24 and other age groups showed that the former was significantly lower than all other age groups (*p* < 0.01). Moreover, low scores were associated with cases where the highest education level of mothers was junior high or high school (*M* = 30.1 ± 6.9, *p* = 0.024), and where childcare or kindergarten was not being utilised (*M* = 30.7 ± 6.5, *p* = 0.029). In addition, scale scores were low for mothers who were not financially comfortable (*M* = 30.4 ± 6.4, *p* = 0.002), and where neighbourly interaction was non‐apparent (*M* = 28.9 ± 6.3, *p* < 0.001).

**TABLE 2:** Relationship between social connectivity of mother and socioeconomic characteristics (*N* = 510)

Items	*n*	(%)	Social conectivity	*p*
			Mean ± *SD*	
Age				
20–24 years	10	2.0	24.1 ± 5.9	0.005
25–29 years	56	11.0	31.9 ± 7.9	
30–34 years	190	37.3	31.1 ± 6.5	
35–39 years	174	34.1	31.9 ± 6.0	
Over 40 years	80	15.7	32.3 ± 6.9	
Partnered				
Yes	495	97.1	31.5 ± 6.6	0.676
No	15	2.9	30.8 ± 6.6	
Employment status				
Working	196	38.4	32.1 ± 6.9	0.285
Parental leave	76	15.5	30.9 ± 6.9	
Homemaker	235	46.1	31.2 ± 6.3	
Highest level of education				
University/Graduate School	184	36.1	32.2 ± 6.4	0.024
Junior/Technical College	203	39.8	31.7 ± 6.6	
Junior High School/High School	123	24.1	30.1 ± 6.9	
Number of children				
1	239	46.9	31.3 ± 7.2	0.456
More than 2	271	53.1	31.7 ± 6.1	
Years of child‐rearing experience (age of oldest child)				
Less than 1 year	55	10.8	31.9 ± 7.6	0.612
More than 1 year	455	89.2	31.5 ± 6.5	
Use of Child care/Kindergarten				
Yes	303	59.4	32.0 ± 6.7	0.029
No	207	40.6	30.7 ± 6.5	
Family structure				
Nuclear family	479	93.9	31.5 ± 6.6	0.920
Extended family	31	6.1	31.4 ± 6.7	
Years of residence at current residence				
Less than 1 year	82	16.1	31.1 ± 7.0	0.547
More than 1 year	428	83.9	31.6 ± 6.6	
Type of housing				
Multi‐dwelling Unit	297	58.2	31.1 ± 6.8	0.105
Single‐detached Dwelliing	213	41.8	32.1 ± 6.4	
Family finances				
Financially comfortable	290	56.9	32.3 ± 6.7	0.002
Not financially comfortable	220	43.1	30.4 ± 6.4	
Neighbourly ties within the neighbourhood				
Apparent	315	61.8	33.1 ± 6.3	<0.001
Not apparent	195	38.2	28.9 ± 6.3	

Note

One‐way ANOVA/*t*‐test.

Table 3 shows the results of four model using the social connectivity of mothers as the dependent variable in a multiple regression analysis. The power of test at this sample size was 0.68.

**TABLE 3:** Results of multiple regression analysis with social connectivity of mother as the dependent variable (*N* = 510)

Items	Model 1				Model 2				Model 3				Model 4			
	β	B	95%CI	*p*	β	B	95%CI	*p*	β	B	95%CI	*p*	β	B	95%CI	*p*
Age																
20–24 years	−0.16	−7.55	−11.66 to −3.44	<0.001	−0.15	−6.91	−11.03 to −2.78	0.001	−0.14	−6.59	−10.70 to −2.48	0.002	−0.12	−5.88	−9.81 to −1.94	0.004
Highest level of education																
Junior high School/High School					−0.09	−1.39	−2.74 to −0.05	0.042	−0.07	−1.14	−2.48 to 0.21	0.098	−0.07	−1.03	−2.32 to 0.26	0.117
Use of Childcare/Kindergarten																
No					−0.09	−1.23	−2.38 to −0.07	0.037	−0.09	−1.26	−2.41 to −0.11	0.032	−0.09	−1.20	−2.30 to −0.10	0.032
Family finances																
Not financially comfortable									−0.12	−1.59	−2.74 to −0.44	0.007	−0.09	−1.22	−2.33 to −0.11	0.031
Neighbourly ties																
Not apparent													−0.29	−3.90	−5.01 to −2.78	<0.001
*R* ^2^				0.158				0.206				0.237				0.370

Note

Illustrates the respective model according to Multivariate Regression Analysis (forced entry). Every items are analysed using dummy variable.

Model 4 indicated that, compared with mothers who were more than 25 years old, the standardising coefficient beta of the social connectivity of young mothers aged 20–24 was low at −0.12 (*p* = 0.004). In Model 2, mothers whose highest level of education was junior high or high school had lower scale scores of −0.09 (*p* = 0.042). Model 3 with family finance is no longer valid (*p* = 0.098). The scale score for no childcare or kindergarten usage was −0.09 (*p* = 0.032), not financially comfortable was −0.09 (*p* = 0.031), and no apparent neighbourly interaction was −0.29 (*p* < 0.001) in Model 4.

## 4 DISCUSSION

### 4.1 Characteristics of mothers with poor social connectivity

The five aspects highlighted in this study are as follows:

1. Age of 24 or less.
2. Junior high or high school as the highest level of education.
3. Not using childcare or kindergartens.
4. Not feeling financially comfortable.
5. No apparent neighbourhood interactions or perception.

The impact of the mother's impression of neighbourhood interactions was particularly significant. According to Rico‐Uribe et al. (2016), the simple presence of a network might not be enough to provide benefits. Holt‐Lunstad (2018) defines social connection as ‘a sense of connection that results from actual or perceived support or inclusion’. In child‐rearing, cognitive aspects of loneliness, such as ‘I feel lonely despite being with other adults,’ may exist even without structural isolation. As seen above, in public health guidance situations, it is useful for specialist public health nurses to not only approach issues by focusing on mothers and their children but also their relationships; this can be done by orienting the mothers’ interest toward the community and facilitate positive awareness. When mothers are not aware of existing connections, support for raising awareness of their value is useful.

Thompson (2015) identified the importance of connecting informally with people who also contribute to child‐rearing by helping in monitoring the child's well‐being. People connecting informally are known as natural mentors or natural helpers (Cowen et al., 1981; Rhodes et al., 1994) and play an important role in supporting child‐rearing in the community (Kusano et al., 2010). Interestingly, Strange et al. (2014) reported that as mothers forge new relationships by interacting within the community, they become sensitised to the importance of building mutually beneficial relationships and having a sense of responsibility to the community. Thus, mothers may potentially develop their connective abilities through their interactions with people within the community. Specialists working to develop community care, such as public health nurses, should strive to build a warm community that welcomes mothers with low connective ability, rather than just providing individual care.

This study found that the younger generation of mothers, those aged between 20 and 24 years, are less able to connect with local people. Women of this age group are still engaged in education or beginning their working lives, and there are few networks of mothers from this age group involved in child‐rearing. Moreover, the average age of women at the birth of first child is now 30.7 years (Japan Cabinet Office, 2019a), leading to an almost 10 years’ age difference between them and the generation of the youngest child‐rearing mothers in the community. Given the circumstances, younger mothers need a higher ability to connect, yet what is seen is that this generation of mothers has low social connectivity, and run a high risk of isolation. The results of this study suggest that support for young pregnant women needs to be expanded, not only to those in their teens but also to those in their early twenties.

Mothers whose highest level of education was junior high or high school showed low scores in Model 2. In other words, mothers with low education often face challenges in connecting with people. Previous studies have also indicated that low education levels are linked to loneliness (Cohen‐Mansfield et al., 2016). This may not be just a matter of academic ability, but also indicative of the impact of poor experiences of interacting with others during important periods in adolescence. Compared with the relatively narrow and fixed communities of life and school up until junior high and high school, where human interaction experiences are primarily with same‐age peers, post‐high school communities are broader, with a greater freedom of choice. Post‐high school, many individuals leave their parental homes to begin life in new areas; the difference in life experiences is likely to affect the development of connecting ability in their later life.

Meanwhile, Laugen et al. (2016) suggested that the provision of intensive support can significantly improve child‐rearing outcomes, even for mothers with education levels at or below high school. Similarly, it would be possible for mothers to gain connections with people through a warm and gentle approach from public health nurses and neighbours.

The results also suggest that mothers who did not use childcare or kindergarten had a low ability to connect. Increased involvement with staff during pick‐up and drop‐off times and among parents occurs when children attend childcare or kindergarten regularly. Experiences such as these are likely to improve a mother's social connectivity. However, there is a dearth of empirical research on changes in parental social connectedness with the start of children's schooling. In October 2019, the Japanese government began offering free preschools and kindergartens (Japan Cabinet Office, 2019b). It is possible that the success of these measures is reflected in outcomes related to maternal connectivity.

In addition, the findings suggest that mothers who answered they were not financially comfortable had low connectivity. According to Roubinov and Boyce (2017), parents’ economic characteristics have a direct effect on their social networks. As a result, the effects of inequality are felt across various social opportunities (Carter, 2018). In addition, unstable household finances reduce mothers’ confidence in child‐rearing (Maehara et al., 2016). In network research, economic characteristics are described as upstream determinants that should be dealt with in sociocultural contexts (Berkman & Krishna, 2014). In Model 3, which considers family finances, the effect of educational background disappeared due to this interaction, thereby indicating the possibility of mothers being able to overcome their social disadvantage with financial support. As economic disparities increase (Japan Ministry of Health, Labour and Welfare, 2017), a radical approach from this upstream economic policy perspective is necessary to foster connectivity between mothers and their communities.

### 4.2 Strengths and limitations

According to the Annual Report on the declining birth rate 2019 (Japan Cabinet Office, 2019a), the average maternal age at birth of first child was 30.7; it was 32.5 for the second child. In this study, mothers aged 30–34 were the largest group. In this regard, the age structure of the study matched that of the Japanese national average.

However, the study has a few limitations. The current research was a cross‐sectional study, so we cannot assert evidence of a causal relationship. The effective response rate in this study was as low as 51.6%. Many of the surveys in Japan utilise opportunities for infant health check‐ups, including face‐to‐face interactions, thereby securing a high response rate. The current low response rate could have been influenced by the fact that a round‐trip mail survey was used. It is also possible that the responses are biased towards cases with good connections. In addition, there are no responses from the teens, and the percentage of respondents in their 20s is low, so the statistical analysis is limited. However, the strength of this study is that several factors have been confirmed when the power of test is not high. In the future studies, it will be worth refining the hypotheses and trying out structural equation modelling.

Further, the results of this survey were obtained from one municipality in Japan. Regional characteristics may have been a mediating factor affecting the richness of maternal social connections.

## 5 CONCLUSION

The most notable characteristic of mothers with low social connectivity with the community was their perception that neighbourly interactions were not apparent. Other important characteristics included maternal age in the early twenties, highest level of education being junior high or high school, non‐use of childcare or kindergarten, and not being financially comfortable.

The findings of this study can inform policymaking, and thus, contribute to the promotion of better academic education, granting subsidies for childcare or kindergartens, addressing economic disparities to aid future mothers, and providing care by community workers to improve mothers’ social connectivity. In addition, community leaders who promote community development can also use the findings to share with other members to highlight how important it is to foster rich human relationships in their communities.

## CONFLICT OF INTEREST

The authors have no conflict of interest to declare.

## AUTHOR CONTRIBUTIONS

Both authors of the manuscript have approved it and agreed with its submission to the journal *Health and Social Care in the community*. H.H contributed to the study concept, design, data collection, analysis, writing this manuscript and acquisition of the Grant in Aid. T.K. assisted with data collection, interpretation of the data, and contributed to the writing of this manuscript.

## Data Availability

The data that support the findings of this study are available on request from the corresponding author. The data are not publicly available due to privacy or ethical restrictions.
